# RAG-Enhanced Open SLMs for Hypertension Management Chatbots

**DOI:** 10.1007/s10916-025-02297-7

**Published:** 2025-11-13

**Authors:** Gianluca Aguzzi, Matteo Magnini, Aqila Farahmand, Stefano Ferretti, Martino Francesco Pengo, Sara Montagna

**Affiliations:** 1 Department of Computer Science and Engineering, University of Bologna, Cesena, Italy; 2 Department of Pure and Applied Sciences, University of Urbino, Urbino, Italy; 3 School of Medicine and Surgery, University of Milano-Bicocca, Milan, Italy; 4 Istituto Auxologico Italiano IRCCS, Milano, Milan, Italy

**Keywords:** Chronic disease self-management, Hypertension, Large language models, Retrieval-augmented generation

## Abstract

Chronic disease management requires continuous monitoring, lifestyle modification and therapy adherence, thus requiring constant support from healthcare professionals. Chatbots have proven to be a promising approach for engaging patients in managing their health condition at home and for offering continuous assistance by being readily available to answer questions. While large language models offer an impressive solution for chatbot implementation, third-party systems raise privacy concerns, and computational requirements limit small-scale deployment. We address these challenges by developing a chatbot for hypertensive patients based on open-source small language models (SLMs), specifically designed for running on personal resource-constrained devices and for providing assistance in QA tasks. In order to guarantee comparable conversational performances with respect to larger language models, we exploited retrieval-augmented generation (RAG) with a local knowledge base. This ensures data privacy by deploying models locally while achieving competitive accuracy and maintaining low computational costs suitable for end-user devices. We experimented with eight SLMs, two prompt configurations, and different RAG strategies – both in the embedding and retrieval components – to identify the most effective solution. The evaluation of our solution grounds on both reference metrics and expert evaluation. Our findings suggest that RAG-enhanced SLMs can improve response clarity and content accuracy. However, our results also indicate that newer SLMs like Qwen3 demonstrate strong performance even without RAG, suggesting a potential shift in the necessity for complex retrieval mechanisms with rapidly evolving model architectures.

## Introduction

Chronic disease management introduces several challenges for healthcare systems and causes significant burdens in patients. Conditions like hypertension require continuous monitoring, prescription adherence, lifestyle adjustments and frequent interaction with healthcare professionals to ensure compliance and maintain patient motivation. The adoption of chatbots, grounded on the latest advancements in language models and specifically designed to support patients in the self-management of chronic conditions, has the potential to become an integral part of the care pathway, complementing clinical practice by providing continuous, accessible support between medical visits. In [1, 2], several attempts are documented: they employ large language models (LLMs) to develop chatbots with diverse objectives, including summarising evidence and providing health advice on screening, diagnosis, treatment, and support for disease prevention.

However, specifically in the context of patient self-management, some critical requirements emerge. First, the interaction between the patient and the chatbot must be as empathetic and anthropomorphic as possible to ensure patients remain motivated and engaged in managing their condition [3–5]. On this respect, evidence found in the literature [6, 7] suggests that different LLMs demonstrate aspects of empathy. At the same time, the information provided by the chatbot must be clear, actionable and highly accurate to effectively support non-expert end users. Accordingly, the risks of hallucinations is not tolerated, as there is no healthcare professional directly mediating the conversation. Moreover, given that patients are likely to share personal health data during these interactions, the chatbot must comply with strong *data privacy regulations*, which precludes the use of third-party systems for natural language processing (NLP) (e.g., GPT-*, Gemini, Claude, etc.) that may store sensitive information. For instance, most of the work reviewed in [1] relies on external third-party LLMs, thus rising privacy and security concerns. Finally, deploying healthcare chatbots requires consideration of computational efficiency: smaller language models, that can run on limited hardware resources while maintaining acceptable response times, are preferable for practical implementation in clinical settings. These small language models (SLMs) offer reduced latency and computational costs, making them more suitable for deployment in resource-constrained environments while still maintaining sufficient performance for patient interactions [8].

In [9], we presented a chatbot designed to support hypertensive patients by providing *timely*, *accurate*, and *empathetic* guidance. The proposed solution evaluated different LLMs (particularly, GPT-3.5 Turbo, Llama2, Alfred, Mistral), both proprietary and open-source, and compared two architectures, each designed to ensure privacy compliance. In the case of proprietary models, a filter for sensitive information was employed. Since, in that experiments, models from the GPT family have demonstrated to be more effective in various tasks compared to the LLMs available at the time, in [10] we conducted experiments aimed at enhancing the performance of open-source LLMs by exploiting retrieval augmented generation (RAG) techniques [11], particularly in question-answering (QA) tasks. To avoid reliance on proprietary third-party services and to address the computational cost – particularly in a design that also considers the possibility of on-device deployment – this paper extends the work presented in [10] by evaluating how the integration of RAG affects the performance of open SLMs in the same QA tasks. This approach involved constructing a knowledge base of QA pairs by collecting data from medical professionals and subsequently enriching this dataset using LLMs to generate additional training examples. Our findings indicate that RAG generally improves response quality over SLM-only baselines, with significant gains for some models (e.g., Gemma 3). However, for newer architectures such as Qwen 3, improvements are smaller and often not significant on our limited dataset, as these models already perform strongly with full-context prompts.

The remainder of this paper is organised as follows. “Background and Motivation” section provides background information and motivation for our work in the context of chronic disease management and the development of a chatbot for hypertensive patients; “Methodology” section describes the methods used in our study, including the RAG technique, and details the dataset used for our experiments; “Results” section presents the results of our study and discusses the implications of our findings; and “Discussion” and “Conclusions” section discusses the results, identifying limitations and strengths, and concludes the paper with a summary of our work and suggestions for future research.

## Background and Motivation

LLM applications in the healthcare field attracted in the last few years several research efforts devoted to experiment advantages and challenges of their adoption as a tool to support, for instance, researchers in the wide spectrum of medical fields, students in their medical education, medical doctors in the definition of diagnosis and therapies and patients in managing their healthcare conditions [12].

In this study, we focus on the use of language models (LMs) as an integral part of a chatbot designed for supporting hypertensive patients in QA interactions. This chatbot is designed to collect hypertension parameters, motivate patients with periodic messages suggesting healthy lifestyle changes, and assist them with any concerns related to their chronic condition [9].

However, some requirements emerge for such an application:

1. The system should communicate empathetically, motivating the patient, making them feel heard, and providing ongoing real-time support [3–6];
2. The system must be highly reliable, with no hallucinations or erroneous information: before deploying LMs in real-world medical environments, it is essential to ensure that models designed for healthcare are accurate, unbiased, and safe for patient use [13];
3. Ethical concerns, including risks of privacy and security [14] must be addressed: *(i)* third-party technology, such as ChatGPT and Gemini, carries an inherent risk of compromising patient privacy, when patients enter test results, ask for support and more. All of this vital health information is potentially collected and stored, potentially compromising patient privacy; *(ii)* the use of open LMs stored on servers still poses risks of data leakage and is not suitable in contexts with limited internet access;
4. The deployment of open-source LMs on the edge, especially if large, presents challenges, primarily due to their hardware requirements. These include sufficient CPU and GPU capabilities, adequate RAM, storage capacity for model weights, and an appropriate operating system environment.

Many of today’s top-performing LMs are proprietary models with hundreds of billions of parameters trained on vast amounts of data. Due to the third and forth requirements, the immediate choice falls on open-source SLMs that can be deployed locally. This approach enables self-hosted deployment on modest computational infrastructure, or even on personal devices alone, thereby addressing privacy concerns by keeping all data processing on-premises, and reduces computational costs compared to large-scale models [15]. Moreover, leveraging locally deployed models provides greater stability over time, as remote services often change their APIs, deprecate models, or shut down entirely, which can disrupt applications that rely on them. For what it concerns the first requirement, there is a growing body of literature focused on the capabilities of LMs in terms of exhibiting empathy [7]. Although there is still room for further evaluation, the consistently high ratings reported in literature [6], suggest that we may consider empathy, in a first approximation, ensured *by design* if models are properly instructed. On the contrary, performances in terms of trustworthiness and accuracy of the answer generated is still subject to evaluation, especially once comparing smaller models with bigger ones. Accordingly, to address the second requirements, the literature suggests two primary techniques: *(i)* fine-tuning and *(ii)* querying local databases to complete specific tasks through RAG.

Fine-tuning refers to training methods applied to general-purpose pre-trained language models for diverse downstream medical applications exploiting the related medical corpus. The RAG model represents a significant alternative, enabling the integration of information retrieval and generative models, allowing access to a specific medical knowledge base without the need of retraining the language models. Both the approaches are recommended to improve model performance and enhance the conversational experience using a domain-specific dataset, ensuring both reliability and empathy in patient interactions. The specific goal is to emulate the language and support typically provided by a healthcare professional.

The literature discussing the application of these techniques in healthcare is rich of examples. In this paper we focus on those devoted to evaluate the system capabilities in QA tasks, namely in analysing the impact of these approaches in providing more accurate and factual answers to medical questions. For instance, [16] presents a SOTA fine-tuning approach that, for the open-source LLMs evaluated, outperforms zero- and few- shot approaches in the QA task. Similar results were obtained with the Scalable and Task-Adaptive Fine-tuning presented in [17] and applied to Llama 2-7B. On the other side, RAG has been exploited for ensuring compliance relevant national guidelines once deploying clinical decision support systems in diverse context. For example, [18] introduces a new LLM framework that combines clinical guidelines with RAG to enhance text interpretation for managing Hepatitis C Virus infection. The findings indicate that this integrated framework outperforms the baseline LLM GPT-4 Turbo model in delivering precise, guideline-specific recommendations. It is worth noting, however, that the landscape is rapidly evolving in this context. Although the referenced papers are recent and their results promising, demonstrating that the application of both fine-tuning and RAG results in improved models, they rely on now-outdated language models (e.g., Llama 2). In light of the rapid release of new language models by both industry and research institutions, the reported results may no longer reflect the current state of the art.

Accordingly, in this paper we focus on specifically experimenting RAG for the QA task as a tool behind a chatbot designed to be deployed on the edge and for supporting specifically hypertensive patients self-management, comparing results obtained with diverse SLMs. In particular, while fine-tuning represents another approach to improve LLM performance, we opted for RAG for several key reasons: (i) Our dataset size is insufficient for effective fine-tuning without risking overfitting, (ii) RAG offers greater transparency by explicitly linking responses to source documents, (iii) It enables dynamic updates to the knowledge base without requiring model retraining, and (iv) It is more computationally efficient, requiring fewer resources than the extensive GPU capacity needed for fine-tuning specialised medical models.

## Methodology

This section outlines our approach to designing and evaluating the RAG system tailored to support hypertensive patients. Our methodology follows a structured pipeline guided by the core design principles outlined in “Background and Motivation” section: privacy preservation and reliable communication. We organise our workflow into five main phases:

1. data preparation (“Data Preparation” section),
2. embedding generation, retrieval and augmentation (“Embedding Generation, Retrieval and Augmentation” section)
3. response generation (“Generation” section), and
4. comprehensive evaluation of the RAG-based system, including a comparative analysis against SLM-only baselines (“Evaluation” section).

**Fig. 1:**
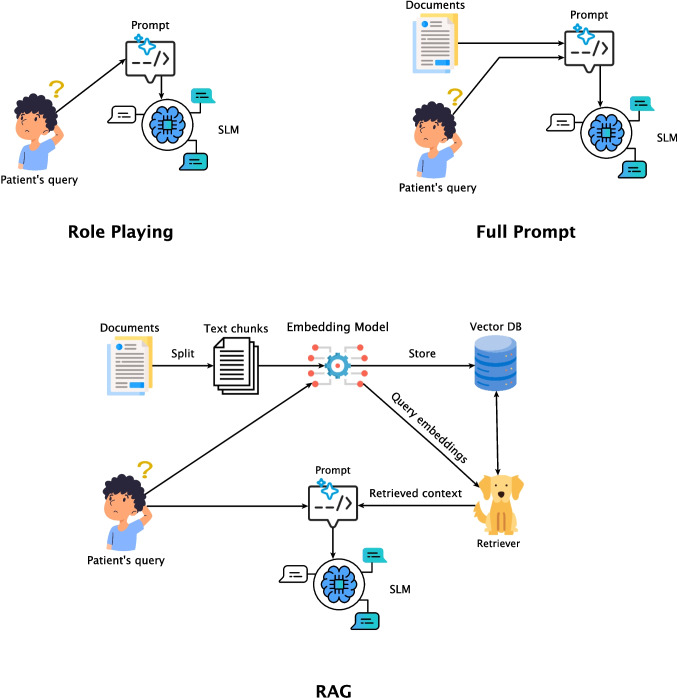
The three different workflows used in the experiments. *Role Playing* is a simple prompting strategy that uses a static prompt with instructions to the model. *Full Context* adds to the static prompt the full content of the documents. *RAG* exploits different retrieval strategies to select the most relevant information to be included in the prompt

An overview of the workflow is illustrated in Fig. 1, while the whole codebase of our experiments is publicly available on Github.^1^

**Fig. 2:**
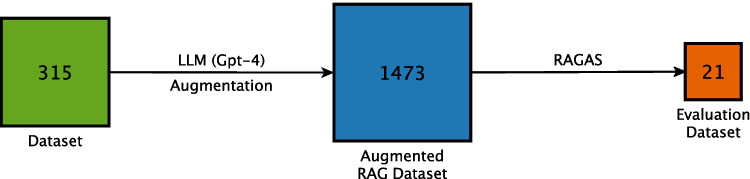
Dataset workflow. The initial dataset is augmented to create the RAG dataset. From this, a subset of 21 samples is extracted using RAGAS to construct the evaluation dataset. Numbers in the boxes represent the number of records in each dataset

### Data Preparation

The dataset is built on several human-to-physician conversations, primarily addressing hypertension, with some discussion of general health topics. Each record consists of a user query and the corresponding response that the chatbot is expected to generate, extracted from a previous work of us [9]. The initial set of 315 entries was expanded using a large language model (GPT-4), resulting in an augmented dataset of 1473 records (see Fig. 2).

#### Augmentation Process

**Fig. 3:**
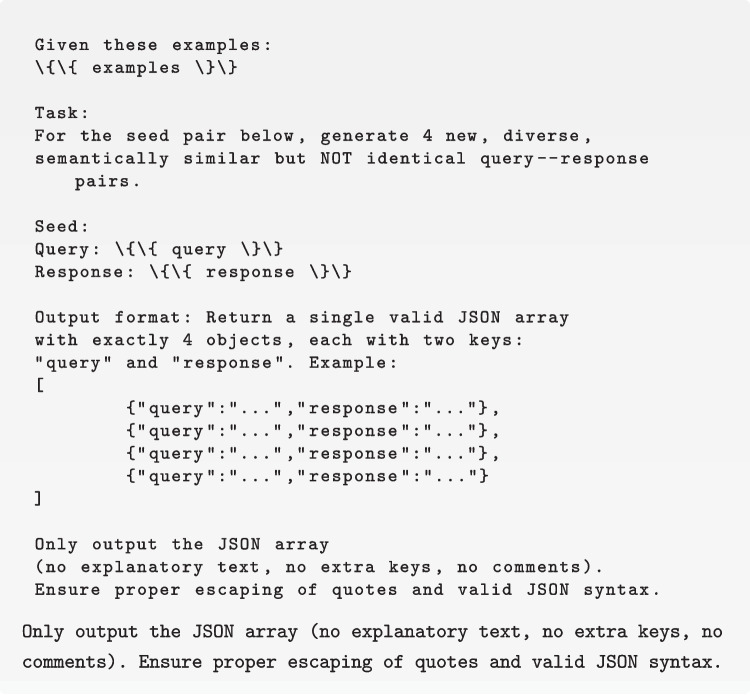
Prompt template used for data augmentation (GPT-4)

This augmentation was performed to transfer the intrinsic knowledge of large language models to smaller ones. Specifically, for each original record we used GPT-4 to generate four additional query—response pairs that were semantically aligned with the seed but phrased differently. To promote diversity and avoid near duplicates, the prompt included all pairs already generated for that record. We repeated this procedure across the corpus until the target size was reached. The prompt structure is shown in Fig. 3.

To ensure the quality of the augmented dataset, we manually reviewed a random sample of 100 records, finding no significant issues. All newly generated pairs were stored alongside their source record, keeping semantically related items adjacent in the dataset.

The average lengths of the query and the response in the augmented dataset are 69.8±14 and 234.8±89 characters, respectively. It is important to note that these dimensions reflect the conversational nature of our domain-specific dataset, where queries represent patient questions (typically brief and focused) and responses mirror the concise yet comprehensive guidance provided by healthcare professionals in clinical consultations. While individual records are relatively compact compared to traditional document corpora, this characteristic aligns with the intended use case of supporting brief, targeted patient-clinician interactions rather than extensive medical literature retrieval.

#### Evaluation Dataset

Finally, to evaluate the chatbot’s performance, a test set from the augmented dataset using the RAGAS^2^ framework. This approach ensured our evaluation dataset effectively covered the diverse query patterns and knowledge domains present in our complete dataset.

RAGAS generates synthetic test sets through a two-phase pipeline: knowledge graph construction and scenario-based query synthesis. This approach addresses the challenge of creating realistic evaluation datasets that mirror production query patterns without requiring extensive manual annotation.

In the first phase, documents are converted into nodes within a knowledge graph—a structured representation where each node contains document content and metadata as properties. The graph is then enriched through three types of transformations: *(i)* splitters perform hierarchical content subdivision (e.g., splitting documents into semantically coherent chunks to enable fine-grained retrieval scenarios), *(ii)* extractors leverage LLMs to identify and extract summaries, key phrases, headlines, and entities from document content, and *(iii)* relationship builders construct inter-node connections using similarity measures such as Jaccard similarity over extracted entities or cosine similarity over embedding representations. This enrichment process creates a semantically interconnected knowledge graph that captures both explicit content relationships and implicit semantic associations.

The second phase employs query-type-specific synthesizers, that are specialized components that generate test samples according to a predefined distribution across four categories representing different cognitive demands: single-hop specific, single-hop abstract, multi-hop specific, and multi-hop abstract queries. Single-hop queries require evidence extraction from one source document (e.g., “What is the normal blood pressure range?”), while multi-hop queries necessitate reasoning and information synthesis across multiple nodes (e.g., “How do lifestyle modifications and medication interact in hypertension management?”). Specific variants target concrete, factual information retrieval, whereas abstract variants require interpretive synthesis and higher-level conceptual understanding. For each generated scenario – a structured specification describing the query type, target nodes, and contextual constraints – synthesizers use LLMs to create plausible user queries grounded in the selected node set, identify the minimal reference contexts (i.e., the smallest subset of documents necessary for faithful answering), and produce ground-truth reference responses. This process ensures that each test sample includes not only a realistic query but also the provenance information needed for retrieval evaluation and the expected answer for generation assessment.

We performed test set generation from our augmented dataset, experimenting with candidate-set sizes from 10 to 40 and settling on a 21-item subset that preserved coverage across the query type distribution while maintaining manageable evaluation complexity. Finally, we manually reviewed all selected queries to confirm their medical relevance and appropriateness for hypertension management contexts.

### Embedding Generation, Retrieval and Augmentation

We generate embeddings based on the augmented RAG dataset using two state-of-the-art models: Nomic [19] (nomic-embed-text-v1.5), and Mxbai^3^ (mxbai-embed-large-v1).

After preprocessing the documents and generating their corresponding embeddings, the resulting text–along with their vector representations and associated metadata–are stored in a vector database using Chroma DB.^4^ The retrieval and augmentation process in our RAG pipeline is designed to enhance the language model’s ability to generate accurate and contextually relevant responses. When a user query is issued it is first converted into a vector representation using the same embedding model previously employed to embed the documents. Subsequently, the retrieval mechanism defined in the RAG pipeline identifies and extracts the top-$K$ most semantically relevant documents from the vector database. This is performed using a similarity search based on cosine similarity, that is computed as:

1 $$\begin{aligned} {\text {cosine\_similarity}}(q, d) = \frac{q \cdot d}{\Vert q\Vert \Vert d\Vert } = \frac{\sum _{i=1}^{n} q_i \cdot d_i}{\sqrt{\sum _{i=1}^{n} q_i^2} \sqrt{\sum _{i=1}^{n} d_i^2}} \end{aligned}$$

Where $q$ is the query vector, $d$ is the document vector, and $n$ is the number of dimensions in the vector space. This range from -1 to 1, where 1 indicates perfect similarity, 0 indicates no similarity, and -1 indicates perfect dissimilarity (namely, the vectors are pointing in opposite directions). However, alternative retrieval methods exist beyond semantic similarity, such as keyword-based approaches like BM25 [20]. As an extension of the TF-IDF (Term Frequency–Inverse Document Frequency) model, BM25 is a sparse retrieval method that scores documents based on the frequency of query terms and their rarity across the corpus, without capturing deeper semantic meaning.

The BM25 ranking method is defined as follows:

2 $$\begin{aligned} {\text {BM25}}(q, d) = \sum _{i=1}^{n} \frac{f(q_i, d) \cdot (k_1 + 1)}{f(q_i, d) + k_1 \cdot (1 - b + b \cdot \frac{|d|}{avgdl})} \end{aligned}$$

where $f(q_i, d)$ is the frequency of term $q_i$ in document $d$, $k_1$ and $b$ are hyperparameters that control the term frequency saturation and document length normalization, respectively, and $avgdl$ is the average document length in the collection.

Once these documents are retrieved (using either semantic similarity or keyword-based search), they are appended to the original user query to form an enriched prompt, which is then passed to the SLMs for final response generation. This retrieval and augmentation process provides the language model with task-specific context and domain-relevant information, enabling it to generate responses that are both accurate and contextually appropriate.

#### Improving Retrieval

In addition to dense semantic similarity-based retrieval (*vector_search*) and sparse keyword-based retrieval (*BM25*) method, we implemented advanced retrieval techniques aimed at enhancing both the relevance and scope of retrieved documents. These methods were evaluated for their effect on the quality of the final responses, with a particular focus on information relevance and contextual accuracy.

##### Hybrid Search

We combined semantic similarity-based retrieval with keyword-based (BM25) retrieval using QueryFusionRetriever from LlamaIndex. This hybrid approach leverages semantic similarity and lexical overlap, weighted by a tunable parameter $\alpha$:

3 $$\begin{aligned} \text {score}_{\text {hybrid}}(q, d_i) = \alpha \cdot \text {sim}_{\text {vec}}(q, d_i) + (1 - \alpha ) \cdot \text {sim}_{\text {bm25}}(q, d_i) \end{aligned}$$

where $\text {sim}_{\text {vec}}(q, d_i)$ is the cosine similarity score from Eq. 1 and $\text {sim}_{\text {bm25}}(q, d_i)$ is the BM25 score from Eq. 2.

##### Reranking

We used an LLMRerank module to refine the list of retrieved documents by reranking top-$k$ documents via LLM scoring function, the role of reranking is to reorder these documents so that the most relevant ones are prioritized for use by the language model, due to the fact that the LLM can better understand the context and relevance of the documents. This can be computed as follows:

4 $$\begin{aligned} R_k=\text {TopK}_{d_i}(\text {sim}_{\text {vec}}(q, d_i)) \end{aligned}$$

5 $$\begin{aligned} \text {score}_{\text {rerank}}(q, d_i)=\text {LLM}_{\text {score}}(q, d_i), \quad \forall d_i \in R_k \end{aligned}$$

6 $$\begin{aligned} \text {Ranked}_k=\text {Sort}(R_k, \text {by } \text {score}_{\text {rerank}}) \end{aligned}$$

Where $R_k$ is the set of top-$k$ documents retrieved by semantic similarity, $\text {LLM}_{\text {score}}(q, d_i)$ is the score assigned by the LLM to each document $d_i$ based on its relevance to the query $q$, and $\text {Ranked}_k$ is the final ordered list of documents that will be used to augment the prompt for response generation. In our experiment, we used the same LLM used for response generation to compute the relevance score, ensuring consistency in the evaluation of document relevance.

### Generation

We explore three primary generation configurations, each with its corresponding prompt structure, as illustrated in Fig. 1, which are described in detail below.

#### SLM-only with Role-playing Prompts

These simulate domain-specific roles (e.g., a medical expert for hypertension) to influence tone and factual precision. System-level instructions enforce empathy (as done in related works [21]), trustworthiness, and Italian-language responses, guiding the SLM to act consistently with a predefined persona. The prompt used was the following: 
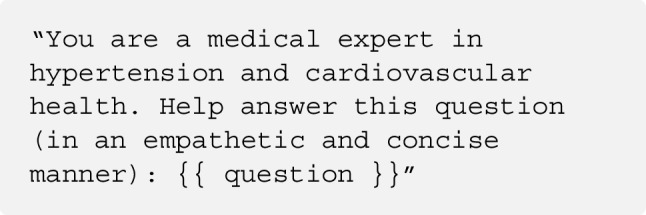


#### SLM-only with Full-context Prompts

The full data-set is embedded within the prompt, and the model is instructed to answer strictly using this context— without referring to prior knowledge. These information-rich instructions provide comprehensive background without retrieval. The prompt in this case is: 
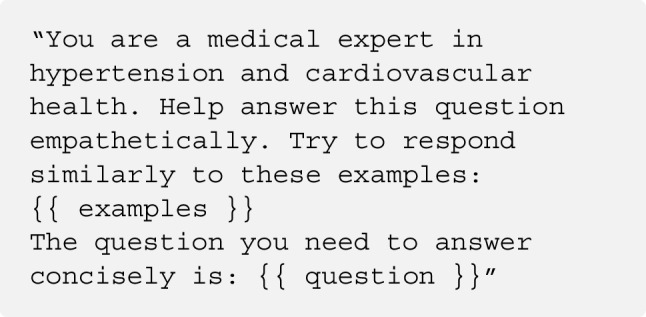


Where the {{examples}} placeholder is replaced with the full content of the documents.

#### RAG-based Generation

Contextual documents, i.e., the top three Q-A pairs according to the retriever’s ranking, are retrieved using various methods to augment the prompt. The prompt template guides a two-stage generation process: (1) an initial response based on retrieved context, and (2) an optional refinement step using additional retrieved information. Refinement logic follows rules such as updating only if new context improves the answer. The base prompt used in this case is:
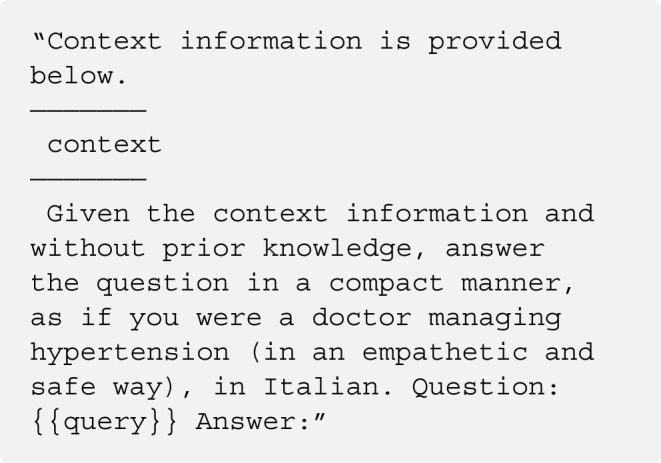


Where the context placeholder is replaced with the retrieved documents and the query placeholder is replaced with the user query. Note that the prompt text shown here has been translated to English for readability, although the original prompts were in Italian—see the code repository for the original prompts.

### Evaluation

Our evaluation phase is designed to assess the contribution of key components in the RAG pipeline, extending beyond the capabilities of traditional prompting approaches. In this regard, we evaluate the overall RAG system’s generative quality compared to SLM-only baselines (both with role-playing and full-context prompts). Although certain configurations, such as the choice of retriever, yielded only marginal performance differences, we include these results for the sake of transparency and to support full reproducibility. We selected a diverse set of SLMs, ranging from recent to older models, to evaluate the impact of the RAG pipeline on the generative quality of the responses. The models used along with the results are detailed in Tables 2 and 3. In order to evaluate both the retrieval and the generative quality of the RAG system, we employ a combination of general-purpose and domain-specific metrics, which are designed to assess the quality of the generated responses in terms of factual accuracy, relevance, and adherence to medical guidelines. In particular, we use the following metrics: faithfulness, and medical faithfulness (G-Eval) [22].

#### Faithfulness

This metric measures how factually consistent a response is with the retrieved context. It ranges from 0 to 1, with higher scores indicating better consistency. The evaluation process involves: (1) extracting all factual claims from the generated response using an LLM which work as a judge, and it is prompted to identify distinct factual statements, (2) systematically verifying each claim against the retrieved contextual documents through semantic matching leveraging again a LLM to determine if the claim is supported by the context, and (3) computing the faithfulness score as the ratio of supported claims to total claims:

$$\text {Faithfulness Score} = \frac{\text {Number of claims supported by retrieved context}}{\text {Total number of claims in the response}}$$

**Table 1:** Key hyperparameters and configurations used in this experiment

Phase	Parameter	Value
Data Preparation	Augmentation Model	Gemini (gpt-4-0613)
Embedding	Embedding Model	mxbai-embed-large-v1
		nomic-embed-text-v1.5
Retrieval	Search Algorithms	Vector Search, BM25, Hybrid Search, Reranker
Retrieval Configurations	Stemmer	Italian Stemmer
	Hybrid Search $\alpha$	0.5
	Reranker Model	qwen2.5:32b
Generation	SLM	smollm2-1.7b, qwen3-0.6b, qwen2.5-0.5b, falcon3-1b, granite3.1-moe:1b, gemma3-1b, llama3.2-1b, deepseek-r1-1.5b
Evaluation	Judge LLM (G-Eval, Faithfulness)	Gemini (gemini-2.5-pro)

This table provides the specific values necessary for reproducing our results

#### Medical Faithfulness (G-Eval)

Because the faithfulness metric only evaluates consistency with the retrieved context and does not capture conformity with medical guidelines or agreement with clinician-authored answers, we complement it with a domain-specific measure based on the G-Eval framework [22]. This G-Eval assessment evaluates medical accuracy, and alignment with expert (physician) responses. The goal is twofold: (1) assess the medical accuracy and safety of generated responses and (2) measure their alignment with clinician-authored answers. We first defined structured evaluation guidelines for medical faithfulness, then used an LLM as an automatic judge to score model outputs. In these guidelines, the following aspects are considered:

- Clinical relevance and linguistic coherence: the response must directly address the question, remain on-topic, and be intelligible.
- Medical accuracy and safety: absence of clinically incorrect, misleading, or potentially harmful statements.
- Precision vs. redundancy: preference for concise, task-focused answers without unnecessary digressions.
- Completeness and expert adequacy: inclusion of all essential information expected from a hypertension specialist, expressed clearly and efficiently.

Responses are rated on an ordinal 1–5 scale: higher scores denote a progression from irrelevant or unintelligible content (1), through topical but clinically unsafe output (2), to mostly correct answers with only minor inaccuracies (3), then fully correct and safe but slightly verbose responses (4), and finally concise, expert, clinically precise, task-appropriate answers (5). For the full prompt (the same given to the physicians) the guidelines are available in Appendix A.

To ensure the complete reproducibility and transparency of our study, we have listed the key hyperparameters and configurations for our experimental setup in a single, comprehensive table in Table 1, which provides all the necessary details for each phase of the experiment, from data preparation to evaluation.

#### Human Evaluation

Two domain experts participated in the evaluation of the 21 QA pairs used in the test. In particular, we collected 189 QA pairs that were selected from the outputs of the three best-performing models, as determined by the automatic computation of the G-Eval metric. Since the goal is to evaluate whether RAG enhances the performance of the model compared to the role-playing and full-context prompts, for each of these three models we selected the answers provided with the two prompts and those provided by the RAG configuration that showed the best G-Eval.

Medical doctors were asked to assess the quality of the answers using a discrete scoring scale ranging from 1 (lowest) to 5 (highest) using the same criteria defined for the G-Eval metric. To avoid bias in the evaluation, no information was provided regarding the model or configuration used to generate each answer.

## Results

We evaluated the performance of different embedding models during the embedding generation phase and conducted a comprehensive assessment of the RAG system’s generative quality. This included comparisons with SLM-only baselines and an analysis of retrieval and augmentation strategies within the RAG pipeline.

**Fig. 4:**
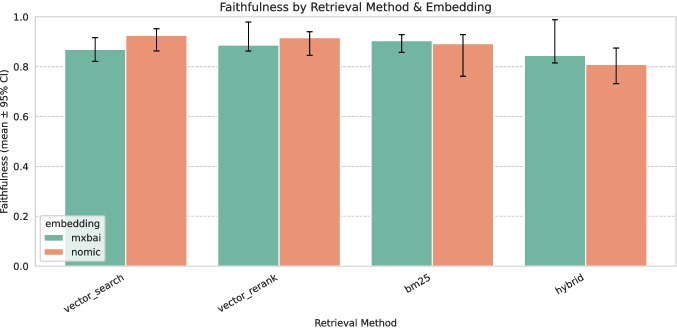
Mean and standard deviation of the faithfulness scores for various embedding models and retrieval configurations

**Fig. 5:**
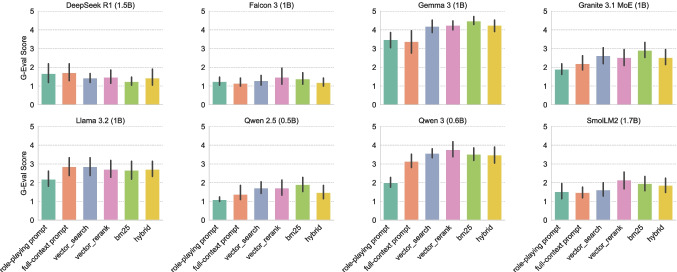
Mean and standard deviation of the G-Eval scores for various RAG methods and SLM-only approaches using the Mxbai embedding model

**Fig. 6:**
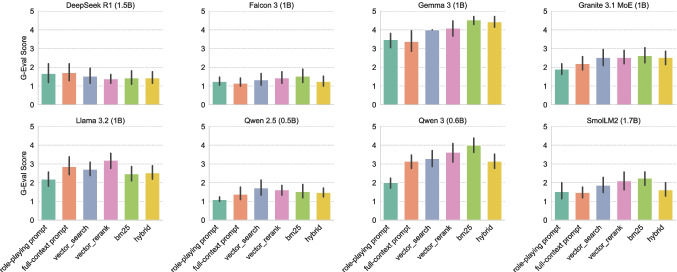
Mean and standard deviation the G-Eval scores for various RAG methods and SLM-only approaches using the Nomic embedding model

**Table 2:** Chi-square test results for RAG vs No-RAG models using *Mxbai* embeddings

Model	G-Eval RAG	G-Eval No-RAG	p-value	Cramér’s V	Significant
DeepSeek R1 (1.5B)	1.48 ± 0.87	1.71 ± 1.10	0.6551	0.196	
Falcon 3 (1B)	1.48 ± 1.08	1.24 ± 0.54	0.1718	0.345	
Gemma 3 (1B)	4.48 ± 0.51	3.48 ± 0.98	**0.0012**	0.616	
Granite 3.1 MoE (1B)	2.90 ± 1.00	2.19 ± 0.87	0.0818	0.400	
Llama 3.2 (1B)	2.86 ± 1.06	2.86 ± 1.15	0.4484	0.297	
Qwen 2.5 (0.5B)	1.90 ± 0.89	1.38 ± 0.92	**0.0404**	0.488	
Qwen 3 (0.6B)	3.76 ± 1.00	3.14 ± 0.91	0.0987	0.387	
SmolLM2 (1.7B)	2.14 ± 1.11	1.52 ± 1.03	0.0779	0.403	

We report the mean and standard deviation of G-Eval scores, the p-value from the chi-square test, and Cramér’s V as a measure of effect size. Significant results ($p < 0.05$) are marked with $\checkmark$ for RAG being statistically better, $\times$ if the null hypothesis cannot be rejected

**Table 3:** Chi-square test results for RAG vs No-RAG models using *Nomic* embeddings

Model	G-Eval RAG	G-Eval No-RAG	p-value	Cramér’s V	Significant
DeepSeek R1 (1.5B)	1.52 ± 0.93	1.71 ± 1.10	0.4219	0.259	
Falcon 3 (1B)	1.52 ± 0.87	1.24 ± 0.54	0.6222	0.205	
Gemma 3 (1B)	4.52 ± 0.51	3.48 ± 0.98	**0.0006**	0.643	
Granite 3.1 MoE (1B)	2.62 ± 0.92	2.19 ± 0.87	0.3006	0.295	
Llama 3.2 (1B)	3.19 ± 0.98	2.86 ± 1.15	0.5333	0.274	
Qwen 2.5 (0.5B)	1.71 ± 0.96	1.38 ± 0.92	0.2191	0.370	
Qwen 3 (0.6B)	4.00 ± 0.95	3.14 ± 0.91	0.0534	0.427	
SmolLM2 (1.7B)	2.24 ± 0.89	1.52 ± 1.03	**0.0022**	0.589	

We report the mean and standard deviation of G-Eval scores, the p-value from the chi-square test, and Cramér’s V as a measure of effect size. Significant results ($p < 0.05$) are marked with $\checkmark$ for RAG being statistically better, $\times$ if the null hypothesis cannot be rejected

### RAG Evaluation

Figure 4 presents the mean and standard deviation faithfulness scores for the various embedding models and retrieval configurations aggregated over all SLMs. Figures 5 and 6 present the mean and standard deviation G-Eval scores for the RAG and SLM-only approaches using Mxbai and Nomic embeddings, respectively. Both Mxbai and Nomic embeddings yield similar results (see Fig. 4), despite the retrival methods showing some differences. Particularly, vector_search performs slightly better in Nomic in terms of faithfulness while bm25 seems to perform better in Mxbai. The hybrid is the worst for both embedders. In general, all the RAG-based approaches outperform the SLM-only baselines (especially in the case of the role-playing prompt). There are some exceptions (e.g., DeepSeek R1 with Mxbai embeddings) but the performance is overall very low for those models, indicating that it is not suitable for our task. It is worth noting that recent models, such as Qwen 3 and Gemma 3, demonstrate strong performance not only with role playing but also with full-context prompts, indicating that newer model architectures are capable of effectively managing longer contexts.

Tables 2 and 3 present the results of the Chi-squared test for independence between the RAG and SLM-only approaches using Mxbai and Nomic embeddings, respectively. The test has been performed among the best performing approach for each group and for each model. Table 2 indicates that there is a statistically significant difference between the best RAG approach (with Mxbai embeddings) and the best SLM-only approach for Gemma 3 and Qwen 2.5 models. Moreover, the Cramér’s V [23] values of 0.616 and 0.488 for these models indicate a strong and moderate association, respectively. In all cases the best RAG approach outperforms or ties with the best SLM-only approach, but the difference is statistically significant only for the two models mentioned above. On the other hand, Table 3 shows that there is statistically significant difference between the best RAG approach (with Nomic embeddings) and the best SLM-only approach for Gemma 3 and SmolLM2. The association is strong for both Gemma 3 – Cramér’s V of 0.643 – and SmolLM2—Cramér’s V of 0.589. Like in the previous case, the best RAG approach outperforms or ties with the best SLM-only approach, but the difference is statistically significant only for the two models mentioned above. Overall, we can observe that the performance of SLMs are indeed positively affected by the RAG pipeline, and for some models the difference is statistically significant.

**Fig. 7:**
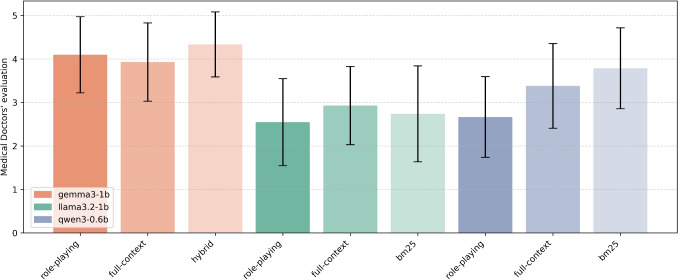
Average scores (on a scale from 1 to 5) assigned by medical experts to the responses generated by the models under the three tested configurations for each model. Standard deviation is indicated by error bars

### Domain Expert Evaluation

In Fig. 7, the results of the medical evaluation are presented as mean ± standard deviation in a box plot, averaged across the evaluations provided by the two physicians and over the 21 QA pairs in 9 different configurations. Physicians’ evaluations largely agree with those obtained using G-Eval. The configuration that yields the best answers is Gemma 3 combined with the hybrid retriever. In two out of the three models, the adoption of RAG improves the answer quality. However, it is difficult to identify a clear pattern where RAG consistently outperforms prompt-only answers. This observation is largely emphasised by Table 4, which reports the results of the Chi-squared test for independence between each pair of configurations chosen within the same LM. In particular, it indicates that there is a statistically significant difference only when comparing the bm25-based RAG approach vs. role playing with the Qwen 3 model. Moreover, for this pair, the associated Cramér’s V value of 0.662 indicates a strong association between the two configurations, suggesting that the observed difference is not only statistically significant but also substantial in terms of effect size. This allows us to conclude that there is a true and meaningful improvement of the bm25 RAG approach compared to role playing with the Qwen 3 model.

**Table 4:** Chi-square test results for each possible pair of methods evaluated by doctors grouped by model

Model	Method 1	Method 2	M-Eval 1	M-Eval 2	p-value	Cramér’s V	Significant
Gemma 3 (1B)	full	hybrid	3.93 ± 0.90	4.33 ± 0.75	0.6767	0.309	
	full	role p.	3.93 ± 0.90	4.10 ± 0.87	0.4047	0.383	
	hybrid	role p.	4.33 ± 0.75	4.10 ± 0.87	0.1919	0.420	
Llama 3.2 (1B)	bm25	full	2.74 ± 1.10	2.93 ± 0.90	0.1875	0.488	
	bm25	role p.	2.74 ± 1.10	2.55 ± 1.00	0.5622	0.372	
	full	role p.	2.93 ± 0.90	2.55 ± 1.00	0.5066	0.355	
Qwen 3 (0.6B)	bm25	full	3.79 ± 0.93	3.38 ± 0.97	0.2845	0.481	
	bm25	role p.	3.79 ± 0.93	2.67 ± 0.93	**0.0102**	0.662	
	full	role p.	3.38 ± 0.97	2.67 ± 0.93	0.3020	0.446	

We report the mean and standard deviation of doctors’ scores, the p-value from the chi-square test, and Cramér’s V as a measure of effect size. Significant results ($p < 0.05$) are marked with $\checkmark$ for RAG being statistically better, $\times$ if the null hypothesis cannot be rejected

## Discussion

This study explored the application of RAG techniques to enhance the performance of open-source SLMs in supporting hypertensive patients. By constructing a domain-specific knowledge base and evaluating various retrieval strategies, we demonstrated that RAG-based systems outperform, in some cases, or match, in most others, with SLM-only baselines in generating accurate and contextually relevant responses.

Moreover, RAG-based approaches proved superior, or equal, in ensuring response quality by evaluating them with G-Eval metric in most of the cases. Notably, RAG solutions that use Mxbai embeddings showed statistical significance in certain scenarios – with Falcon 3 and Granite 3.1 models – further validating their effectiveness in medical applications.

Despite these successes, we acknowledge that recent models, such as Qwen 3 and Gemma 3, achieve state-of-the-art performance in the QA task even without the need for retrieval, also demonstrating strong contextual understanding and response generation capabilities with our full context prompts. This clearly indicates that, in the future, the need for RAG pipelines in local deployments may be reduced, as newer models are capable of effectively managing longer contexts and generating accurate responses without requiring retrieval mechanisms. Moreover, the impressive performance we observe here may provide further evidence that, in the future, instead of relying on large models for all tasks, it is more feasible to employ a set of smaller models that are specialised in specific tasks. This approach offers the potential to significantly reduce computational overhead and the demand for extensive computational resources.

### Limitations

Although the study provides an updated and reproducible assessment of RAG applied to open-source SLMs for hypertension self-management, several limitations constrain the strength and generalisability of the conclusions. We make them explicit to prevent overinterpretation and to guide future work.

#### Limited Evaluation Sample and Statistical Power

Only 21 items were ultimately used for automatic evaluation and statistical testing. This markedly limits statistical power; consequently, the absence of significance for many model/configuration comparisons cannot be interpreted as evidence of equivalence. The current work should be viewed as a pilot investigation that establishes methodological foundations and provides preliminary evidence for RAG effectiveness in this domain, but requires validation on substantially larger, clinically-reviewed test sets to establish robust generalisability claims.

#### Synthetic Data Augmentation Risks

The majority of the augmented corpus (1473 records) was generated with GPT-4. While a random manual spot check of 100 records did not reveal critical errors, no systematic clinical validation of all synthetic entries was performed. This may introduce subtle factual inaccuracies, stylistic homogenisation, or distributional shift, potentially biasing both retrieval and generation performance. The compact size of individual records (averaging 235 characters for responses) reflects the conversational nature of patient-clinician interactions but may limit the complexity of knowledge representation and retrieval scenarios compared to larger document corpora.

#### Single Language and Cultural Scope

All data, prompts, and evaluations are in Italian. This constrains generalisability to other languages, where tokenisation behaviour, idiomatic phrasing, and medical terminology density differ. Cross-lingual robustness (e.g., zero-shot transfer to English / Spanish) and multilingual embedding alignment were not assessed.

#### Absence of Patient-centred Evaluation

Apart from a limited domain expert scoring exercise (Fig. 7), no end-users (patients) participated. We did not measure comprehension, health literacy adaptation, trust, or sustained engagement over time. The chatbot was evaluated only in single-turn QA; multi-turn dialogue coherence, consistency across paraphrased queries, and conversation state handling remain untested.

In summary, while our findings support the feasibility of privacy-preserving RAG combined with SLM pipelines for hypertension QA, the present study should be viewed as an exploratory, methodical baseline rather than a deployment-ready clinical evaluation.

## Conclusions

In this paper, we evaluated privacy-preserving RAG with open SLMs for hypertension self-management, showing that retrieval generally yields equal or better medical faithfulness than SLM-only prompting, while its marginal benefit narrows for the most recent architectures (e.g., Qwen 3). The approach remains relevant for deployment on modest hardware, offering a pragmatic balance between accuracy and data control.

Future work should expand and clinically validate evaluation sets with a pilot study, thus increasing also the statistical power of the results presented in this paper. Moreover, we are planning to move beyond single-turn QA to longitudinal, patient-centred interaction studies with a focus on engagement, safety perception, adherence impact. Finally, in future work we plan to conduct a precise methodological study in order to possibly identify specific categories of questions or scenarios in which RAG provides measurable advantages, as well as those where it may be redundant or counterproductive. To this end, we will focus on expanding the dataset across domains and question types, enabling a more fine-grained and generalisable analysis of the impact of RAG in different contexts.

## Data Availability

No datasets were generated or analysed during the current study.

## Appendix A: Evaluation Criteria

Medical doctors have been asked to evaluate the responses generated by the models following these criteria:

1. — **Off-topic response**: The answer is completely unrelated to the question or is entirely unintelligible or nonsensical in the context of the Italian language. *Example: If asked “What is your name?”, the answer is “18 years old.”*
2. — **Contextually relevant, but factually incorrect and/or potentially harmful**: The response is relevant to the question, but contains incorrect or misleading information that could lead to harmful consequences. *Example: If asked “How is blood pressure measured?”, the answer is “To measure blood pressure, you must insert a needle into the arm.”*
3. — **Substantially correct, with minor inaccuracies**: The response is mostly correct and clinically useful, but includes small errors, negligible inaccuracies, or minor issues such as grammatical mistakes or slightly off-topic content. *Example: If asked “How is blood pressure measured?”, the answer describes the correct method but indicates an obviously incorrect hypertension threshold like “190/20 mmHg.”*
4. — **Correct, but includes superfluous information or lacks clarity**: The answer is accurate and complete, but contains redundant details or non-essential information that an expert would typically omit for the sake of brevity. Alternatively, it may be correct but expressed in a verbose or unclear way. *Example: If asked “How is blood pressure measured?”, the response explains the correct method but includes lengthy paragraphs about diet and general hypertension management.*
5. — **Correct, concise, and effective response**: The answer is precise, to the point, and clearly conveys all necessary information without unnecessary elaboration. This is the type of answer an expert would provide. *Example: A clear, succinct, and accurate explanation of how to measure blood pressure, without digressions.*

## Footnotes

1 https://github.com/Aqila-Farahmand/experiments-2025-joms-rag

2 https://docs.ragas.io/en/stable/

3 https://huggingface.co/mixedbread-ai/mxbai-embed-large-v1

4 https://github.com/chroma-core/chroma
